# Spinal cord infarction presenting as Brown-Séquard syndrome from spontaneous vertebral artery dissection: a case report and literature review

**DOI:** 10.1186/s12883-019-1559-0

**Published:** 2019-12-12

**Authors:** Yang-Yang Meng, Le Dou, Chun-Mei Wang, De-Zheng Kong, Ying Wei, Li-Shan Wu, Yi Yang, Hong-Wei Zhou

**Affiliations:** 1 Department of Radiology, the First Hospital of JiLin University, Xinmin St. #71, Changchun, 130021 China; 2 Department of Neurology, Stroke Center, Neuroscience Center & Clinical Trail and Research Center for Stroke, the First Hospital of JiLin Universit, Xinmin St. #71, Changchun, 130021 China

**Keywords:** Spinal cord infarction, Vertebral artery dissection, Magnetic resonance imaging, Brown-Séquard syndrome

## Abstract

**Background:**

Spinal cord infarction (SCI) is rarely caused by vertebral artery dissection (VAD), which is an important cause of posterior circulation stroke in young and middle-aged patients. We report the case of a middle-aged patient without obvious risk factors for atherosclerosis who had SCI from right VAD.

**Case presentation:**

An otherwise healthy 40-year-old man presented with acute right-sided body weakness. Six days earlier, he had experienced posterior neck pain without obvious inducement. Neurologic examination revealed a right Brown-Séquard syndrome. Magnetic resonance imaging (MRI) of the head was normal. Further, cervical spine MRI showed spinal cord infarction (SCI) on the right at the C1-C3 level. Three-dimensional high-resolution MRI (3D HR-MRI) volumetric isotropic turbo spin echo acquisition (VISTA) scan showed evidence of vertebral artery dissection (VAD). The patient was significantly relieved of symptoms and demonstrated negative imaging findings after therapy with anticoagulation (AC) and antiplatelets (AP) for 3 months.

**Conclusions:**

The possibility of vertebral artery dissection (VAD) should be considered in the case of young and middle-aged patients without obvious risk factors for atherosclerosis. Furthermore the VISTA black blood sequence plays an important role in the pathological diagnosis of vertebral artery stenosis. Early correct diagnosis and active therapy are crucial to the prognosis.

## Background

Spinal cord infarction (SCI) is caused by the decrease of blood flow in feeding arteries or vascular occlusion of the spinal cord, resulting in low perfusion or spinal cord ischemic lesions. Because of extensive vascular collateral network in the spinal cord, which has a high tolerance to ischemia and hypoxia, the incidence of SCI is low. The most common presentation is anterior spinal artery syndrome; therefore, vertebral artery dissection (VAD) resulting in SCI presenting as Brown-Séquard syndrome is even rarer. To the best of our knowledge, only one case has been reported in the literature [1]. VAD is one of the main causes of ischemic stroke in young and middle-aged people. We report a rare case spinal cord infarction presenting as Brown-Séquard syndrome from spontaneous VAD to raise awareness of this condition.

## Case presentation

A 40-year-old man, a building worker, presented with acute right-sided body weakness without obvious inducement for 6 days. Weakness of the right limb occurred 16 h prior to admission to the hospital, presenting as inability to lift the right upper limb and inability to stand on the right lower limb.

There was no history of trauma and neck massage, no surgical history, and no previous infectious symptoms. He did not have diabetes and he was normotensive. The patient was a smoker for 10 years.

Neurologic examination showed that the patient’s consciousness and speech were normal. Cranial nerve examination was normal. According to the Medical Research Council muscle scale, the right upper proximal limb had muscle power grade 4/5; distal limb, muscle power grade 2/5; right lower limb, muscle power grade 0/5; and contralateral upper and lower limbs, muscle power grade 5/5. Joint position and vibration disappeared in the right lower limb, and position was weakened in the right hand. Deep sensation was normal in the left side of the body. The right ankle reflex was weakened. Pain and temperature sensation were decreased on the left below the level of C3. The patient tested negative for the Kernig sign. These results indicated Brown-Séquard syndrome on the right at the level of C3.

Laboratory studies, including hematologic, biochemical, and immunologic investigations were normal. Lumbar puncture cerebrospinal fluid was unremarkable.

There were no obvious abnormalities on head computed tomography (CT) and MRI examinations.

**Fig. 1:**
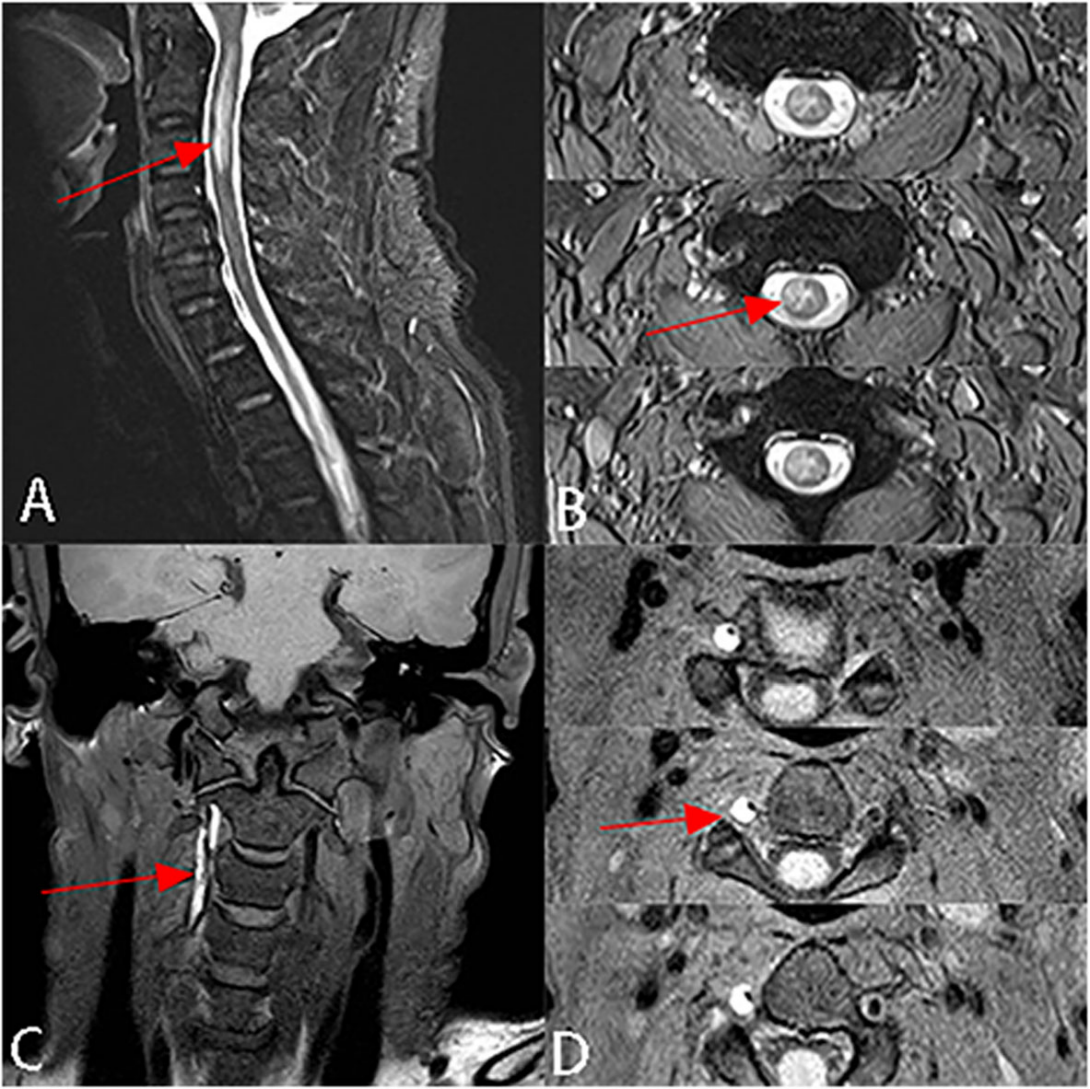
**a** Sagittal T2-weighted magnetic resonance image of cervical spine reveals spinal cord enlargement with hyperintense lesion at the level of C1–3 (red arrow). **b** Axial T2WI shows hyperintense signal of right spinal cord (red arrow). This is consistent with spinal cord infarction (SCI). (**c**)(**d**) HR-MRI VISTA sequence shows narrowing of the right vertebral artery at the level of C1–3 with eccentric high signal parallel to the narrowed lumen (red arrow)

Sagittal T2-weighted MRI of cervical spine revealed spinal cord swelling with hyperintense lesion at the level of C1–3 (Fig. 1a). T2 axial scan showed hyperintensity of right spinal cord, consistent with SCI (Fig. 1b). Further, high-resolution MRI volumetric isotropic turbo spin echo acquisition (HR-MRI VISTA) showed narrowing of the right vertebral artery at the level of C1–3 with eccentric high signal parallel to the narrowed lumen resulting from VAD with intramural hematoma (Fig. 1c, d). Therefore, we diagnosed this condition as SCI resulting from VAD.

**Fig. 2:**
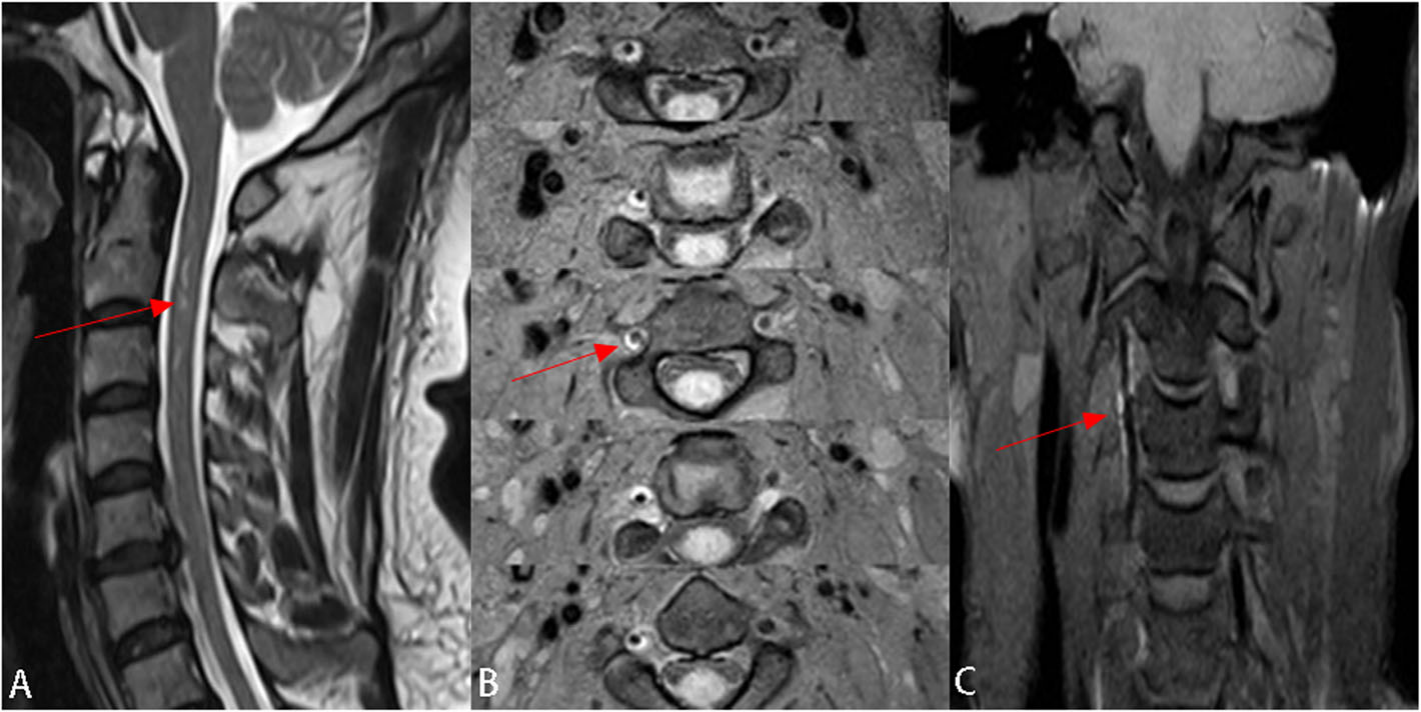
Post-treatment image (**a**) After 3 months of therapy, Sagittal T2-weighted magnetic resonance image of cervical spine shows the range of abnormal signal of spinal cord in level C1–3 diminished. (**b**)(**c**) HR-MRI VISTA reexamination shows that lumen recanalization of the right vertebral artery

The patient underwent anticoagulant (AC) and antiplatelet (AP) therapy for 3 months. MRI reexamination showed a diminished range of abnormal signals of the spinal cord (Fig. 2a), and HR-MR VISTA sequence revealed lumen recanalization of the right vertebral artery (Fig. 2b, c). The patient recovered well and was discharged with a modified Rankin scale score of 1. We advised the patient to keep following up, but he refused.

## Discussion and conclusions

We report a rare case of SCI presenting as Brown-Séquard syndrome caused by VAD in a middle-aged patient. The incidence of SCI is much lower than that of cerebral infarction. It constitutes less than 1% of all central nervous system ischemic events [2]. The most frequently affected region is the anterior spinal artery territory, and the thoracic spinal cord is most commonly involved. The most common etiologies include spinal cord trauma, aortic surgery, vascular injury, arterial dissection, thromboembolic disease, atherosclerosis, vasculitis, hypercoagulable states or mass effect on the spinal cord. VAD is not commonly seen, but accounts for approximately 10–25% of ischemic stroke in middle-aged people [3, 4]. The etiology of VAD may be either traumatic or spontaneous [1]. Possible etiologies of spontaneous VAD include infection, smoking, hypertension, autosomal dominant genetic disease, congenital dysplasia of connective syndrome, dysplasia of muscle fibers, etc.

**Fig. 3:**
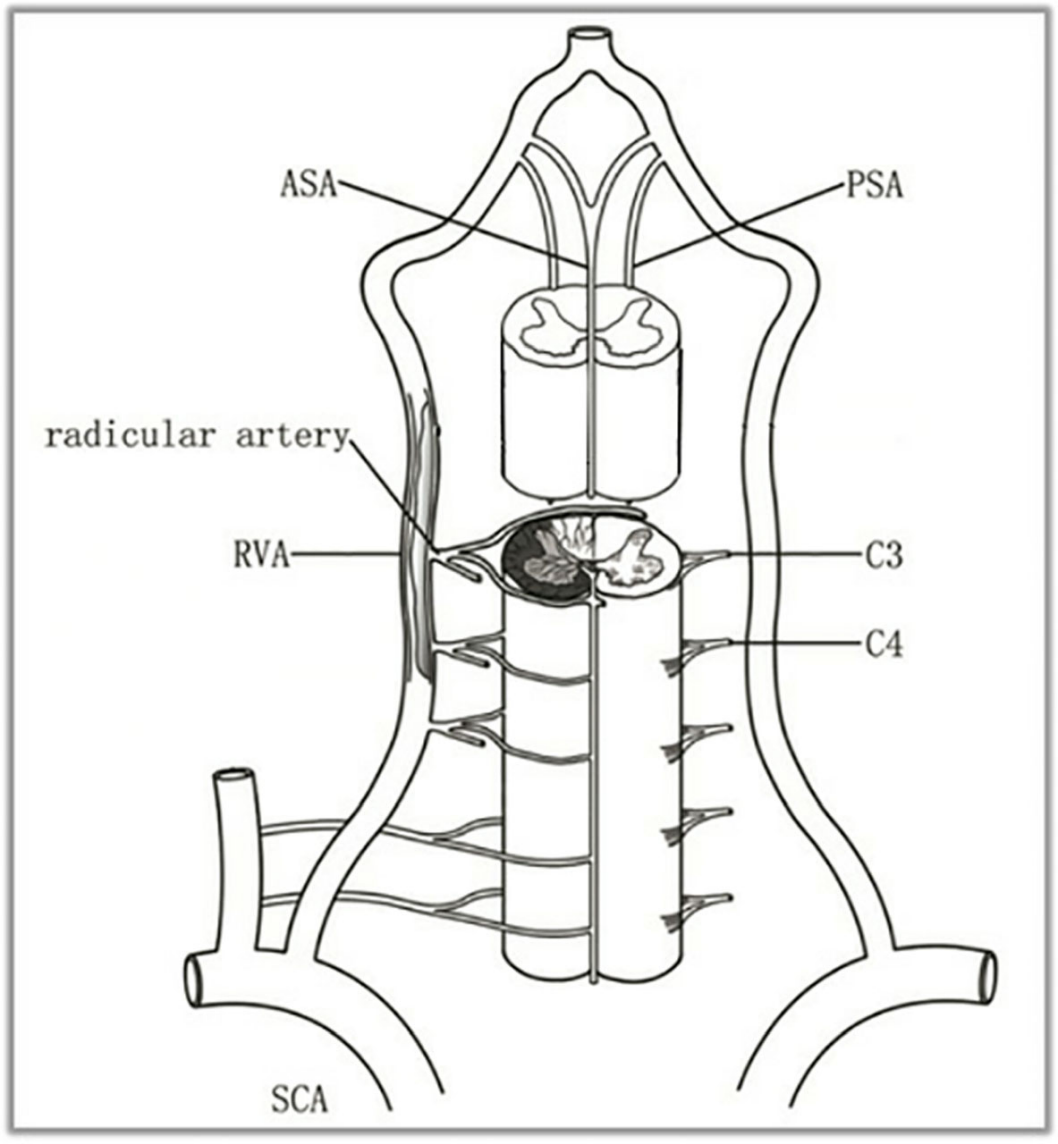
Spinal cord arerial supply. The arteries supplying the spinal cord include the anterior spinal artery (ASA), the posterior spinal artery (PSA), and the radicular artery. The upper region (C1-T2 segments) of the spinal cord is supplied mainly by the vertebral artery. Starting at the level of C3, the anterior and posterior spinal arteries receive a branch from the vertebral artery, that of the radicular artery. In our case, the basic lesion of this patient is the occlusion of the cervical radicular artery caused by vertebral artery dissection, which eventually resulted in right spinal cord infarction

Familiarity with the anatomy of spinal cord arterial supply is a prerequisite for understanding the imaging findings and clinical features of SCI (Fig. 3). The arteries supplying the spinal cord include the anterior spinal artery (ASA), the posterior spinal artery (PSA) and the radicular artery. The ASA supplies the anterior two-third area of the cross-section of the spinal cord. The PSA supplies the posterior column and posterior cord of the spinal cord, which is equivalent to the posterior one-third of the cross-section of the spinal cord. The radicular artery entering the intervertebral foramen is divided into two branches: the anterior root artery and the posterior root artery, which are anastomosed with the ASA and the PSA to form a spinal coronary artery ring, which supplies the surface of the spinal cord and the peripheral region of the parenchyma; ischemia, therefore, does not easily occur in the spinal cord. The radicular artery is supplied by branches from the vertebral artery and the inferior thyroid artery in the cervical segment. As described above, we concluded that the basic lesion of this patient was the occlusion of the cervical radicular artery caused by VAD, which eventually resulted in SCI presenting as Brown-Séquard syndrome.

Clinical manifestations vary with the location of the lesion. Spinal cord syndrome is divided into five main categories: anterior cord syndrome, posterior cord syndrome, central cord syndrome, Brown-Séquard syndrome, and cauda equine syndrome [5]. Anterior cord syndrome is the most common, while Brown-Séquard syndrome such as in our patient, is rare, with a constellation of ipsilateral signs of posterior column and pyramidal tract dysfunction, with contralateral loss of pain and temperature due to involvement of the lateral spinothalamic tract [4]. As far as we know, there have been few reports of VAD presenting as Brown-Séquard syndrome.

MRI is the most important diagnostic tool to evaluate spinal cord syndromes because it can clearly display the structure of the spinal cord and directly identify the location of the lesion. The location of the abnormal signal is consistent with the area where the blood vessels supply that region of the spinal cord. The classic imaging finding of SCI is that T2WI shows hyperintense lesion in areas with diseased vascular supply. HR-MRI, VISTA sequence can be used for large-scale collection of images of the head and neck, with black blood imaging effect, and can be post-processed in any direction by three-dimensional reconstruction, so as to evaluate the pathological changes of the vessel wall and lumen, thus it can assist to differentiate various vasculopathies [6]. In addition, the VISTA sequence was superior to magnetic resonance angiography for the intramural hematoma of VAD, which was characterized as high-signal intramural hematoma distributed along the blood vessels. To the best of our knowledge, there has been little literature reporting the use of the VISTA black blood sequence for the diagnosis of VAD. In our case, VISTA black blood sequence showed eccentric high signals parallel to the narrowed lumen caused by VAD with intramural hematoma, confirming our suspicion of VAD (Fig. 1c, d).

Current recommendations for the management of cervical artery dissection emphasizes the use of antithrombotic therapy for 3 to 6 months [7]. The latest Cervical Artery Dissection in Stroke Study (CADISS) randomized clinical trial shows that there are no differences in prevention of stroke or of residual stenosis and occlusion between patients treated with either AC or AP [8]. In our case, this patient improved after AC and AP therapy for 3 months.

In conclusion, in the case of young and middle-aged patients without obvious risk factors for atherosclerosis, the possibility of VAD should be taken into account. 3D HR-MRI VISTA examination should be further performed to avoid missed diagnosis and misdiagnosis. Early correct diagnosis and active therapy are crucial to the prognosis.

## Data Availability

All data related to this case report are documented within this manuscript.

## Abbreviations

3D HR-MRI: Three-dimensional high-resolution magnetic resonance imaging
AC: Anticoagulation
AP: Antiplatelet
ASA: Anterior spinal artery
CADISS: Cervical Artery Dissection in Stroke Study
CT: Computed tomography
MRI: Magnetic resonance imaging
PSA: Posterior spinal artery
RVA: Right vertebral artery
SCA: Subclavian artery
SCI: Spinal cord infarction
VAD: Vertebral artery dissection
VISTA: Volumetric isotropic turbo spin echo acquisition
